# HIV and syphilis knowledge, perceptions, and practices among Myanmar migrant workers in Samut Sakhon Province, Thailand

**DOI:** 10.1186/s12889-022-14674-9

**Published:** 2022-11-28

**Authors:** Alfrison Paloga, Dumrongkiet Arthan, Pannamas Maneekan, Patreeya Kitcharoen, Apichai Wattanapisit, Chirawat Paratthakonkun, Suparat Phuanukoonnon, Shoon Lei Nyan Wai Tun, Ngamphol Soonthornworasiri

**Affiliations:** 1 Department of Tropical Hygiene, Faculty of Tropical Medicine, Mahidol University, Bangkok, 10400 Thailand; 2 Department of Tropical Nutrition and Food Science, Faculty of Tropical Medicine, Mahidol University, Bangkok, 10400 Thailand; 3 Department of Society and Health, Faculty of Social Sciences and Humanities, Mahidol University, Nakhon Pathom, 73170 Thailand; 4 Department of Clinical Medicine, School of Medicine, Walailak University, Nakhon Si Thammarat, 80160 Thailand; 5 College of Sports Science and Technology, Mahidol University, Nakhon Pathom, 73170 Thailand; 6 Department of Social and Environmental Medicine, Faculty of Tropical Medicine, Mahidol University, Bangkok, 10400 Thailand

**Keywords:** Condoms, HIV, Migrant, Myanmar, Syphilis

## Abstract

**Background:**

Thailand has been one of the largest migration hubs in Southeast Asia for the past four decades and keeps attracting migrants from neighboring countries. Due to difficulties associated with their status, migration can place individuals at a heightened risk for sexually transmitted diseases. This study aimed to examine factors influencing HIV and syphilis preventive behaviors among Myanmar migrants in Samut Sakhon, Thailand.

**Methods:**

A cross-sectional mixed-method study was conducted among Myanmar migrants aged 18 years and above in Samut Sakhon Province, Thailand. To fulfill this study's aims, four hundred seventy-three respondents completed a survey to provide quantitative data, and eight participants completed in-depth qualitative interviews. The factors associated with protective sexual behaviors were identified with multiple logistic regression analysis of the quantitative study data and thematic analysis of the qualitative data.

**Results:**

The respondents showed good knowledge of HIV and syphilis (50.1%), but over half (55.6%) exhibited the negative perception of sexually transmitted disease prevention; about 81.4% of male respondents never used a condom when they had sexual intercourse in the past year. Based on multivariate analysis, income expenses-balance (adjusted odds ratio (AOR) = 2.379, 95% confidence interval (CI): 1.002–5.731, *p* = 0.049), number of sex partners (AOR = 3.044, 95% CI: 1.339–6.924, *p* = 0.008), and having sex with a prostitute (AOR = 6.085, 95% CI: 1.28–28.918, *p* = 0.023) were all statistically associated with unprotected sex. In the qualitative analysis, knowledge, understanding, beliefs; the influence of culture, community, and environment; and condom perceptions were also important factors.

**Conclusion:**

Low use of condoms in sexual practice was identified, and the appropriate intervention or approach to improve the utilization of condoms in the community was provided.

## Background

Over the past four decades, Thailand has been a major destination in Southeast Asia for migrant workers in agriculture, fishery, industry, and hospitality [1]. According to the report of the International Labour Organization (ILO), there were 2,131,751 documented migrant workers in December 2021; 1,715,940 (80.5%) came from Myanmar [2]. Migration can increase the individual’s vulnerability in sexually transmitted infections (STIs) because of the difficulties related to their status [3]; as reported by the UNAIDS in 2014, the prevalence of HIV among migrants from neighboring countries was up to four times higher than the prevalence in general population [4]. Therefore, migrant workers have been recognized as one of the priority groups for HIV prevention, treatment, and care [5]. Similar to the general population, unsafe sex is a leading risk factor of STIs among migrants, and migrants are at a higher risk [6–9].

A number of studies in Thailand revealed that migrant workers engaged in HIV-risk-increasing behaviors such as low rates of condom use [10–12] and frequent visits to sex workers [10, 12]. High prevalence of HIV was reported among Cambodian migrant workers in Trat Province, and a growing number of AIDS cases are also reported in Samut Sakhon Province, a common location of Myanmar migrant workers [13]. Another study among Myanmar migrant workers in Chiang Mai Province discovered an overall HIV-1 prevalence rate of 4.9%, nearly two times higher than in comparable population groups (21-years-old male military and pregnant women) in the province [14]. The Care International Organization, a non-governmental organization in Thailand, reported 1,929 HIV cases in Myanmar migrants between 1998 and 2000 [15]. A study conducted in Phangnga province reported the prevalence of syphilis infection and HIV among migrant workers at 0.44% and 2.28%, respectively [16]. A majority of migrants (90%) did not consider themselves at risk for HIV or STIs, and this perception is related to their knowledge and the educational attainment [10].

Understanding individuals’ knowledge, attitudes, and practices related to HIV, syphilis, and other STIs is essential for developing community-wide STI control and prevention strategies [17, 18]. A study reported a moderate level of knowledge of HIV risk factors among Cambodian and Myanmar migrants in Thailand, and a poor level of knowledge were reported in women, people with low education, seafarers, farmers, and people who did not received information about HIV/AIDS [19]. A study among reproductive-aged Myanmar migrants in Samut Sakhon Province demonstrated the moderate level of basic knowledge of HIV/AIDS with positive perspectives toward HIV/AIDS control and prevention measures related to safe sex [20]. Another study among young Myanmar migrant workers showed moderate STI knowledge and high HIV/AIDS knowledge were associated with already having sexual intercourse; however, the higher knowledge, positive attitudes, and higher barriers to STIs did not associate with having multiple sexual partners and protective sex [21]. Although these studies indicate that Myanmar migrants have a moderate level of knowledge, it is still lower than other ethnic migrants in Thailand [22]. A study reported the insignificant correlation between respondents' knowledge and condom use [23]. However, the understanding of HIV and syphilis preventive behaviors among Myanmar migrants is still unclear.

This study aimed to determine the factors influencing HIV and syphilis preventive behaviors among Myanmar migrants in Samut Sakhon, Thailand, using knowledge, attitudes, and practices as a practical framework for planning community health promotion and STI prevention efforts.

## Methods

### Study area

The study site was Samut Sakhon Province. It is located in a coastal region in central Thailand known as the center of Thailand's seafood industry, which attracts many migrant workers. Furthermore, it is one of Thailand's regions with the highest number of Myanmar migrants [21, 24].

### Study design and context

A cross-sectional mixed-method study with a sequential model design was conducted [25]. The target population was adult Myanmar migrants (age ≥ 18 years) living in Samut Sakhon Province, Thailand. The study was conducted in two phases (February 2020 and October to November 2020) because of COVID-19 pandemic lockdown restrictions.

The sample size was calculated by using Wayne's sample size calculation formula to estimate the infinite population proportion to derive a sample count for this study [26] with the following assumptions; a previous study in Samut Sakhon Province among youth Myanmar migrant workers showed 49.4% had the middle level of knowledge on STI and HIV/AIDS [21], a confidence interval level of 95% and a margin of error of 5%. The total sample was determined to be 461, with an estimated drop-out rate of 20%. However, we recruited 473 respondents who came for routine blood screening at Samut Sakhon hospital and met the selection criteria: Myanmar migrants aged ≥ 18 years.

### Data collection

Convenience sampling was used to select respondents for the quantitative study. Myanmar migrants who came for routine blood screening at the Samut Sakhon Hospital were invited to participate in the study. Participants completed the self-administered questionnaire, and illiterate participants were helped by Myanmar native students from Mahidol University who had already been trained. Our study tool was a self-administered paper-based questionnaire to collect and measure participants' knowledge, attitudes, perceptions, and practices regarding HIV and syphilis. The questionnaire consisted of demographic questions and questions on respondents' basic knowledge about syphilis and HIV, risk factors, prevention, and health beliefs. The questionnaire was developed in English and translated to Myanmar language by a researcher who is a native Myanmar speaker. All the processes and documents (questionnaire, participant information sheet, and informed consent form) were written and explained in the Myanmar language.

Also, we used purposive sampling to select respondents for in-depth qualitative interviews as a part of the study. All interview documents (guidelines, list of questions) were in Myanmar, and a Myanmar native researcher conducted the interviews, taking notes and recording all the interviews to prevent missing key information. The intimate nature of this study's topic required sensitivity and creating a comfortable environment for the interviewees. All respondents were interviewed individually in a private room with only an interviewee and an interviewer inside. The interview could be ended early upon the request by the participant. We conducted a preliminary analysis after each interview. The data saturation was considered if we found the same contents came out repeatedly, and no new information was discovered.

### Data analysis

Two research team members double-entered, cleaned, and checked the qualitative data, and we analyzed the quantitative data using PASW Statistics for Windows, version 18.0 (SPSS Inc., Chicago, IL, USA). We used descriptive statistics for the frequency and percentage distribution of demographic characteristics, basic knowledge about syphilis and HIV, risk factors, prevention, and health beliefs. We also used binary logistic regression to assess the relationships between the independent and outcome variables. We considered *p*-value less than 0.05 statistically significant for the univariate and multivariate analysis models.

For the HIV and syphilis knowledge questions, we gave 1 point for each correct answer and divided it into two levels based on the mean score, poor (lower than the mean score) and good (higher than the mean score) for the analysis. We used items based on the health belief model to measure respondents' positive perceptions toward STI prevention, and the scoring for each item was 1 point for disagreeing, 2 points for a neutral stance, and 3 points for agreeing. For negative perceptions, the score was reversed. Perceptions toward STI prevention was divided based on the mean score for the analysis: low (lower than the mean score) and high (higher than the mean score).

Protective sexual behaviors entailed engaging in less-risky sexual activities, especially consistently using condoms, and these items asked about marital status, sex with others, and condom use. We categorized the married respondents as either 1) unprotected (sex with others without using a condom) or 2) protected (sex with others but using a condom or no sex with others). Next, we categorized the single, widowed, or divorced respondents as 1) unprotected (sex with others who were not regular partners without using a condom) or 2) protected (used a condom during sexual intercourse).

For the qualitative data analysis, the transcripts of the Myanmar interview recordings were back-translated into English to ensure each translation's validity and accuracy for data analysis [27]. Manual thematic analysis was used to analyze the qualitative data [28].

## Results

### Sociodemographic characteristics

Table 1 presents the study participants' sociodemographic data. Out of 473 respondents, 297 (62.8%) were female, and 278 (58.8%) were 25–39 years old. By education level and marital status, most respondents, 226 (47.8%), received a secondary school education, and 345 (72.9%) were married. Burmese was the largest ethnic group, with 386 respondents (81.6%). A total of 357 (75.5%) survey respondents were employed as factory laborers, and 214 (45.2%) thought their income expenses-balance was sufficient. Most workers had health insurance; the social security insurance was the most common health insurance scheme (38.7%). By length of stay in Thailand, 212 (45.9%) respondents had been in the country for one to five years.

**Table 1:** Respondents’ Sociodemographic Characteristics (*n* = 473)

Sociodemographics	n (%)
Sex	
Male	176 (37.2)
Female	297 (62.8)
Age	
18–24	85 (18)
25–39	278 (58.8)
40–59	109 (23)
≥ 60	1 (0.2)
Mean ± SD: 33.05 ± 8.897	
Education	
Illiterate	13 (2.7)
Elementary school	149 (31.5)
Secondary school	226 (47.8)
High school	74 (15.6)
College/University or higher	11 (2.3)
Marital Status	
Single	97 (20.5)
Married	345 (72.9)
Divorced	23 (4.9)
Widow	8 (1.7)
Ethnicity	
Burma	386 (81.6)
Mon	41 (8.7)
Karen	14 (3)
Chin	2 (0.4)
Shan	8 (1.7)
Kayah	2 (0.4)
Rakhine	5 (1.1)
Other	15 (3.2)
Occupation	
Unemployed	44 (9.3)
Construction labor	6 (1.3)
Agriculture labor	7 (1.5)
Restaurant/shopkeeper labor	10 (2.1)
Factory labor	357 (75.5)
Fisherman	35 (7.4)
Housemaid	6 (1.3)
Others	8 (1.7)
Income expenses-balance	
Not enough/debt	91 (19.2)
Sufficient	214 (45.2)
Saving	24 (5.1)
Transfer back to the family	144 (30.4)
Length of stay in Thailand (*n* = 462)	
< 1 year	50 (10.8)
1 – 5 years	212 (45.9)
> 5 years	200 (43.3)
Health insurance	
Social security	183 (38.7)
Health insurance card	150 (31.7)
Private insurance	5 (1.1)
No insurance	135 (28.5)

### Knowledge regarding HIV and syphilis

Regarding the respondents' knowledge of HIV, Table 2 shows that more than half (53.1%) of respondents have a good level of knowledge about HIV, mainly related to the route of transmission and preventive measures. In contrast, for the knowledge of syphilis, 57.3% of respondents showed lower general knowledge of syphilis, and more than 50% of participants answered each question incorrectly.

**Table 2:** Respondents' HIV and Syphilis Knowledge

Knowledge	Correct Answer	
	**n (%)**	
	**HIV**	**Syphilis**
The causal agent of HIV/Syphilis	71 (15)	54 (11.4)
The possibility of being healthy-looking and having HIV/Syphilis	155 (32.8)	171 (36.2)
The drug to cure HIV/Syphilis	132 (27.9)	175 (37)
**Symptoms:**		
Rapid weight loss	271 (57.3)	N/A
Diarrhea that lasts for more than a week	183 (38.7)	N/A
Prolonged swelling of the lymph glands in the armpits, groin, or neck	77 (16.3)	N/A
Sore in the mouth, anus, or genitals	57 (12.1)	N/A
Painless sore in the genital area	N/A	148 (31.3)
Painless sore in the mouth	N/A	139 (29.4)
Painful urination	N/A	36 (7.6)
Pus-like or blood discharge from the genital	N/A	37 (7.8)
Skin rash	N/A	176 (37.2)
**Routes of transmission:**		
Sexual intercourse	344 (72.7)	268 (56.7)
Sharing injection needles	352 (74.4)	N/A
Sharing food/drink	193 (40.8)	N/A
Infected pregnant mother to baby	338 (71.5)	227 (48)
Kissing or physical contact	150 (31.7)	131 (27.7)
Infected blood transfusion	369 (78)	N/A
Sharing underwear/towel/clothes	N/A	83 (17.5)
**Preventive measures:**		
Being faithful to your partner	263 (55.6)	N/A
Taking contraceptive pills	142 (30)	129 (27.3)
Using a condom every time during sex	278 (58.8)	227 (48)
Showering or cleaning genitals after having sex	80 (16.9)	70 (14.8)
Using gel or Vaseline when having sex	N/A	79 (16.7)
**Level of knowledge (*n* = 473)**	**n (%)**	
**HIV**		
Poor	222 (46.9)	
Good	251 (53.1)	
Mean ± SD: 7.3 ± 3.872		
**Syphilis**		
Poor	271 (57.3)	
Good	202 (42.7)	
Mean ± SD: 4.55 ± 3.505		
**Overall knowledge**		
Poor	236 (49.9)	
Good	237 (50.1)	
Mean ± SD: 11.85 ± 6.621		

### Perceptions of HIV and syphilis disease and prevention

Table 3 shows the results of the participants' perceptions toward STI prevention based on the health belief model components, which are perceived susceptibility, perceived severity, perceived benefits, and perceived barriers. Concerning perceived susceptibility, more than half of the respondents had sex without a condom, however, most respondents disagreed that they were at risk of HIV or syphilis. Concerning perceived severity, most respondents agreed that HIV and/or syphilis were severe diseases with tremendous negative impacts on their and others' lives.

**Table 3:** Respondents' Perceptions Toward the Sexual Transmitted Diseases Prevention (health belief model)

Health Belief Model	Agree	Neutral	Disagree
	**(%)**	**(%)**	**(%)**
**Perceived susceptibility**			
If I had sex without a condom, I would get syphilis	67	21.2	11.9
I only had oral sex; I will not get syphilis	36.2	37.1	26.7
If there is someone diagnosed with syphilis at my house, I think I also can get syphilis	47.8	24.9	27.2
I think I am at risk of getting syphilis	31.3	23.2	45.4
If I had sex without a condom, I would get HIV	75.1	15.9	9
I only had oral sex; I will not get HIV	39.1	31.9	29
If there is someone diagnosed with HIV at my house,	54.5	16.8	28.7
I think I also can get HIV	33.3	17.7	49
I think I am at risk of getting HIV	67	21.2	11.9
**Perceived severity**			
If I had HIV, my life would end	60.3	17.4	22.3
If I had HIV, my partner also would get the disease	76.8	13.9	9.3
If I / my wife had HIV during pregnancy/breastfeeding, can pass HIV to my baby	80	14.2	5.8
If I had HIV, my family and I would be discriminated by others	74.2	15.4	10.4
If I had syphilis, my partner also would get the disease	65.5	20.6	13.9
If I / my wife had syphilis during pregnancy, it can cause problems to my baby	71.6	21.7	6.7
**Perceived benefits**			
Consistent and correct condom use can prevent syphilis	73	22.6	4.3
Consistent and correct condom use can prevent HIV	78.3	14.8	7
Consistent and correct condom use can prevent pregnancy	77.4	15.7	7
**Perceived barriers**			
Condom make sex less fun/pleasure interference	35.7	49.6	14.8
Latex condom and the lubricant used in it make me allergic/itching	28.4	55.4	16.2
Partner refuses to use a condom	24.3	48.7	27
Feeling shy to buy or ask for a condom	47.2	31.9	20.9
Need extra money to buy a condom	42.9	29.3	27.8
Feeling shy to buy or ask for a condom	35.7	49.6	14.8
Need extra money to buy a condom	28.4	55.4	16.2
**Level of perception toward the sexual transmitted diseases prevention (*n* = 473)**	n (%)		
Low	263 (55.6)		
High	210 (44.4)		
Mean ± SD: 47.17 ± 3.965			

Most respondents agreed on the benefits of consistent and correct condom use as a contraceptive method and a barrier against HIV and syphilis infection. However, in response to the perceived barriers to condom use, feeling too shy to buy or ask for a condom was the main barrier to their use.

### Risk behaviors

Table 4 presents the data on the respondents' sexual behaviors related to risk, protection, and preventive measures. A total of 402 respondents (85%) previously had sexual intercourse: 241 (62.9%) had sex for the first time between the age of 18 and 24, 364 (90.5%) only ever had one sexual partner, and 307 (76.4%) never consumed alcohol before intercourse. Four (1%) used dating apps to find a sexual partner, 23 (5.7%) previously had a same-sex experience, and 9 (2.2%) admitted at least once having sex with a prostitute; of the latter group, only 1 (11.1%) always used a condom.

**Table 4:** Respondents' Risk Factors, Preventive Practices, and Protective Sexual Behaviors (*n* = 402)

Risk factors	Male n(%)	Female n(%)	Total n(%)
Age first time had sex (*n* = 383)			
12–17	7 (5.3)	25 (10)	32 (8.4)
18–24	79 (59.8)	162 (64.5)	241 (62.9)
25–39	45 (34.1)	64 (25.5)	109 (28.5)
40–59	1 (0.8)	0 (0)	1 (0.3)
Sexual partner			
One	123 (87.9)	241 (92)	364 (90.5)
Two	13 (9.3)	14 (5.3)	27 (6.7)
> two	4 (2.9)	7 (2.7)	11 (2.7)
Ever consumed alcohol before sex			
Always	7 (5)	3 (1.1)	10 (2.5)
Sometimes	58 (41.4)	27 (10.3)	85 (21.1)
Never	75 (53.6)	232 (88.5)	307 (76.4)
Used dating apps to find a sexual partner			
Yes	1 (0.7)	3 (1.1)	4 (1)
No	139 (99.3)	259 (98.9)	398 (99)
Ever had the same-sex experience:			
Yes	9 (6.4)	14 (5.3)	23 (5.7)
No	131 (93.6)	248 (94.7)	379 (94.3)
Ever had sex with a prostitute:			
Yes	4 (2.9)	5 (1.9)	9 (2.2)
No	136 (97.1)	257 (98.1)	393 (97.8)
Use a condom when having sex with a prostitute: (*n* = 9)			
Always	1 (25)	0 (0)	1 (11.1)
Sometimes	3 (75)	1 (20)	4 (44.4)
Never	0 (0)	4 (80)	4 (44.4)
**Preventive practice**			
Protection with spouse			
None	116 (65.9)	239 (80.5)	355 (88.3)
Condom	21 (11.9)	20 (6.7)	41 (10.2)
Others	3 (1.7)	3 (1)	6 (1.5)
Ever had sex with others			
Yes	15 (10.7)	8 (3.1)	23 (5.7)
No	125 (89.3)	254 (96.9)	379 (94.3)
Protection with others: (*n* = 23)			
None	6 (40)	3 (37.5)	9 (39.2)
Condom	9 (60)	5 (62.5)	14 (60.8)
Others	0 (0)	0 (0)	0 (0)
The reason for using protection^a^:			
Prevent infection from/to partner	8 (80)	5 (100)	13 (92.86)
Contraception	2 (20)	0 (0)	2 (14.3)
Others	0 (0)	0 (0)	0 (0)
Used a condom when having intercourse during the last year			
Every time	11 (7.9)	11 (4.2)	22 (5.5)
Sometimes	15 (10.7)	12 (4.6)	27 (6.7)
Never	114 (81.4)	239 (91.2)	353 (87.8)
Easy to access a condom			
Yes	49 (35)	58 (22.1)	107 (26.6)
No	40 (28.6)	87 (33.2)	127 (31.6)
Not sure	51 (36.4)	117 (44.7)	168 (41.8)
How usually get a condom^a^:			
Buy from pharmacy	14 (38.9)	14 (41.2)	28 (57.1)
Buy from store	7 (19.4)	5 (14.7)	12 (24.5)
Buy from the vending machine	0 (0)	2 (5.9)	2 (4.1)
Free distribution from NGO	5 (13.9)	5 (14.7)	10 (20.4)
Free distribution from the health facility	9 (25)	8 (23.5)	17 (34.7)
Other	1 (2.8)	0 (0)	1 (2)
**Protective sexual behavior**			
Unprotected			59 (14.7)
Protected			343 (85.3)

^a^*Multiple responses*

Of the 402 respondents who previously had sexual intercourse, 379 (94.3%) had sex with their spouse or regular partner, and 355 (88.3%) did not use protection. Twenty-three (5.7%) had sex with others, and 9 (39.2%) did not use protection. Regarding condoms, 353 (87.8%) of the respondents never used a condom during the last year, and 168 (41.8%) were unsure whether obtaining condoms was easy, although 57.1% reported usually buying condoms from a pharmacy (Table 4).

### Protective sexual behaviors

We measured the respondents' protective sexual behaviors, the outcome variable of this study, by combining the scores for the questions about ever having sex with others with those for questions about consistent condom use and analyzing them based on marital status. Because this area of the study related to sexual behavior, we only included the 402 respondents who had previously ever had sex; of them, 343 (85.3%) were engaging in protective behaviors (Table 4). Table 5 shows the multivariate analysis findings for these 402 respondents. We found that income expenses-balance, number of sex partners, and having sex with a prostitute were all statistically associated with protective sexual behaviors (*p* < 0.05).

**Table 5:** Multivariate Analysis of All Factors and Protective Sexual Behaviors

Factors	Protective sexual behavior		Univariate		Multivariate	
	**Unprotected** **(*n* = 59)**	**Protected** **(*n* = 343)**	**Crude OR** **(95% CI)**	***p*-value**	**Adjusted OR** **(95% CI)**	***p*-value**
Sex						
Male	23 (39)	117 (34.1)	1		1	
Female	36 (61)	226 (65.9)	0.81 (0.459–1.431)	0.469	0.933 (0.484–1.8)	0.837
Age Group						
< 40	42 (71.2)	255 (74.3)	1		1	
≥ 40	17 (28.8)	88 (25.7)	1.173 (0.635–2.166)	0.61	1.167 (0.601–2.266)	0.649
Education Level						
Low Level	20 (33.9)	128 (37.3)	1		1	
High Level	39 (66.1)	215 (62.7)	1.161 (0.649–2.077)	0.615	1.063 (0.568–1.99)	0.848
Income expenses-balance						
Not enough	7 (11.9)	77 (22.4)	1		1	
Enough	52 (88.1)	266 (77.6)	2.15 (0.939–4.926)	0.07	2.379 (1.002–5.731)	0.049*
Health Insurance						
No Insurance	18 (30.5)	98 (28.6)	1		1	
Have Insurance	41 (69.5)	245 (71.4)	0.911 (0.499–1.663)	0.762	1.06 (0.549–2.046)	0.862
Knowledge						
Poor	28 (47.5)	170 (49.6)	1		1	
Good	31 (52.5)	173 (50.4)	1.088 (0.626–1.892)	0.765	0.9876 (0.539–1.804)	0.963
Perception						
Low	29 (49.2)	192 (56)	1		1	
High	30 (50.8)	151 (44)	1.315 (0.757–2.287)	0.331	1.573 (0.853–2.901)	0.146
Sex Partner						
1	46 (78)	318 (92.7)	1		1	
≥ 2	13 (22)	25 (7.3)	3.595 (1.718–7.52)	0.001*	3.044 (1.339–6.924)	0.008*
Alcohol before sex						
No	42 (71.2)	265 (77.3)	1		1	
Yes	17 (28.8)	78 (22.7)	1.375 (0.742–2.55)	0.312	1.044 (0.505–2.155)	0.908
Same-sex experience						
No	54 (91.5)	325 (94.8)	1		1	
Yes	5 (8.5)	18 (5.2)	1.672 (0.596–4.691)	0.329	1.207 (0.355–4.101)	0.763
Sex with prostitute						
No	54 (91.5)	339 (98.8)	1		1	
Yes	5 (8.5)	4 (1.2)	7.847 (2.043–30.143)	0.003*	6.085 (1.28–28.918)	0.023*

*OR* Odds ratio, *CI* Confidence interval, *p < 0.05

Based on the significantly associated variables, respondents who believed their income expenses-balance were sufficiently demonstrated a 2.379 times higher chance of engaging in unprotected sexual conduct (adjusted odds ratio, AOR, = 2.379, 95% confidence interval, CI: 1.002–5.731, *p* = 0.049) than those for respondents whose income expenses-balance were insufficient. For those with ≥ 2 sex partners, the odds of unprotected sex were 3.044 times (AOR = 3.044, 95% CI: 1.339–6.924, *p* = 0.008) greater than those for respondents who only had one sex partner. Furthermore, respondents who had sex with a prostitute demonstrated 6.085 times higher chance of engaging in unprotected sex (AOR = 6.085, 95% CI: 1.28–28.918, *p* = 0.023) than those respondents who had not.

### Qualitative results

The Myanmar native member of the research team conducted in-depth interviews with eight Myanmar migrants to explore more about their HIV and syphilis risk perceptions. There were seven women and one man, six were between 31 and 40 years old, and two were between 41 and 50 years old. Two were factory workers, four were housewives, one was a shopkeeper, and one was unemployed.

We grouped the interview responses data into three thematic categories: general knowledge understanding, perceptions, and beliefs; culture, community, and environmental influences; and condom perceptions.

### General knowledge, understanding, and beliefs about HIV and syphilis

For most interviewees, fear came to their minds when they heard about HIV and syphilis. Generally, their knowledge was limited to modes of transmission. They perceived a high risk of HIV and other STIs from sexual misbehavior, particularly having sex outside of wedlock, such as with a prostitute: "you can get HIV because of cheating on your spouse" (female, housewife, 31–40 years).

All participants were aware of HIV, but only a few had heard about syphilis. All believed these diseases could be transmitted to others in one's household by living in the same house, sharing food and clothes, and doing activities together. The participants perceived HIV infection as conferring double jeopardy because, in addition to suffering themselves, patients harmed their family members significantly. One respondent even said, "if I got HIV, I would stay away from the family, and then I would commit suicide because I [would be] afraid the family will get the infection from me" (female, unemployed, 41–50 years).

Myanmar migrants exhibited close kin relationships in their communities and upheld their traditions and culture. Growing up in Myanmar, these migrants greatly influenced local traditional values and taboos. Most participants reported no differences between their sexual behaviors in Thailand and Myanmar. In Myanmar, they lived among and were surrounded by family members who would know if one did something wrong, and this kind of culture still exists even though they live in Thailand. They lived together in tight-knit migrant communities where everyone knew each other, and there was peer pressure to behave properly. Therefore, Myanmar cultures such as living among groups or relatives, virginity, and monogamy are the things they still keep in mind. For instance, a participant who resided in Thailand for more than eight years stated, "it is not acceptable to have more than one wife when you already got married" (female, housewife, 41–50 years). Showed that monogamy was the societal norm held even among the immigrant communities in Thailand.

However, slight differences were found between the older and younger generations. Cultural differences between Thailand and Myanmar influenced the younger Myanmar generations. Thai society is more open-minded, especially about same-sex relationships, and this societal environment influenced the younger generation to be more accepting of different sexual preferences. Not all respondents considered this a positive development:

It [premarital and same-gender sex] has become common now. When they come here alone to work, they get more freedom, and even more from online social media using mobile phones nowadays. I noticed that social media, especially in Thailand, is so easily accessible to all kinds of information, but if it becomes too much freedom without control, I do not like it. (male, factory worker, 31–40 years).

In addition to the generational differences, grounded in the technological advances that increased the ease of information sharing and communication. One participant described how technology was changing young peoples' sexual behaviors:

The young generation was not shy and more likely to make physical contact with their boyfriend or girlfriend or even have sex before marriage. It has become common in big cities because of what they learn and see from online social media using mobile phones. (female, housewife, 31–40 years).

### Understanding and perceptions of condoms

Contraception was not new among the Myanmar migrants in this study; they reported knowing about contraceptive measures and even using them before they came to Thailand. However, most respondents only mentioned pills and injections as contraception options. In Myanmar, the condom was a relatively new invention, and condoms were difficult to obtain outside of towns and cities, where they could be purchased at pharmacies and grocery stores; in rural areas, condoms were only available through health facilities.

The interviewees discussed taboos attached to condom use, such as that condoms were only necessary to prevent STIs from sex outside of wedlock, such as with prostitutes. Also, people thought condoms were only for unmarried couples to prevent pregnancy. Since the monogamy was well accepted and highly valued, using condom in the close-knit community would rather be unacceptable. Therefore, most respondents said they would not use a condom:

I think condoms are not as good contraception as pills. For disease prevention, I have never needed to use it since I only have my wife as one sexual partner, and my wife has already used contraception. (male, factory worker, 31–40 years).

Generally, these Myanmar migrant interviewees reported limited access to condoms but also demonstrated negative images of their use, and perceiving shame in being seen to buy condoms because of the implied promiscuous behavior which is unacceptable behavior.

## Discussion

Through this study, we identified the perceptions, beliefs, knowledge, and practices related to HIV and syphilis in particular and about risky and protective sexual behaviors in general with respect to STI transmission among a group of Myanmar migrants living in Thailand. We found low to moderate knowledge of information about HIV and syphilis, fairly negative views on condom use, and adequate knowledge of at least some risky and some protective behaviors, including the commitment to one's partner and consistent condom use.

### Knowledge of protective sexual behavior

Half of the participants demonstrated good general knowledge and were less likely to engage in unprotected sexual behavior in this study. However, we found no significant correlation between knowledge and protective sexual behavior. This contradicted the findings of Nyunt et al. (2008), who found that Myanmar migrants' knowledge was substantially associated with safe sex behavior in Samut Sakhon [29]. Another study [30] indicated that Myanmar migrants in Thailand demonstrated little understanding of HIV, but those authors detected no significant link with safe sex practices. Moreover, regarding HIV and syphilis knowledge, we found that respondents knew more about HIV than syphilis; more than half of the respondents answered every syphilis question incorrectly. Additionally, respondents talked more about HIV than syphilis during the in-depth interviews.

In addition, we identified that participants demonstrated an incorrect understanding of routes of HIV transmission, and several believed that HIV might be spread through sharing food or physical contact. Some interviewees stated that if they contracted HIV, they would remain away from their family for fear of infecting family members. Not only in this study but also the previous studies among Myanmar migrants in Tak Province [11], Pathumthani Province [30], Samut Sakhon Province [23], and the latest study in Surat Thani Province [31] also found the same result: incorrect understandings or misunderstandings are still prevalent in the Myanmar migrant community. Therefore, a health intervention to increase understanding of HIV and syphilis is required in this community.

Epistemology defines knowledge as a comprehension process influenced by truth, belief, and justification; it divides knowledge into three productive phases: knowing that, knowing how, and fully understanding why [32]. The respondents in this study appear to still be in the phase of knowing that but not fully comprehending it. They believe they live in a safe environment because HIV and syphilis are inconspicuous and because they have never seen, been, or known anyone close to someone diagnosed with HIV or syphilis, which could be one of the factors. Data from Samut Sakhon Hospital showed the prevalence of HIV among Myanmar migrant workers in the study area around 0.2% [33], which is low compared to the prevalence of HIV among Burmese migrants workers in Chiang Mai Province [14], and Migrants workers in Phangnga Province [16] were 4.9% and 2.28%, respectively.

### Perceptions of protective sexual behavior

In this survey, more than half of the participants perceived sexually transmitted disease prevention poorly. Although no significant link was found between perceptions and protective sexual behavior, individuals with high perceptions of HIV and syphilis disease and prevention were more likely to engage in unprotected sex. This result was found to be contradictory to a study by Nyunt et al. (2008) that showed a significant association between high perceptions of safe sex practices and actual safe sex behaviors [29], and another study in Ranong Province that also found negative attitude was statistically associated with unsafe sex [34]. Some factors that might contribute to our conflicting results include knowledge and partner trust.

Some misunderstandings contribute to individuals' sexual behaviors [35] as infected people look unhealthy. Only one-third of the participants in this study correctly identified that one can look healthy while presenting with HIV and syphilis, and one-third believed they could not contact HIV or syphilis through only oral sex. Separately, most respondents in this study were married, which is relevant to partner trust. Married and cohabiting partners tend to trust their partners regarding STI risk and regarding sex with others in general, which relates to condom use [8, 36]. In this study, two-thirds of the married respondents did not think they were personally susceptible to HIV or syphilis infection.

Also, we established respondents' perceptions of the benefits of protective sex practices. More than 70% agreed on the benefits of consistent and correct condom use for contraception and STI prevention, but we also identified some barriers. Primarily, the study interviewees cited being shy about buying, asking, or asking for a condom. In the in-depth interviews, some respondents emphasized the negative perception of condoms in Myanmar communities as only being for unmarried couples and men who are having sex with someone other than their wives. These perceptions supported the findings from a study about gender equality and cultural norms in Myanmar that sex is a taboo topic, and the Myanmar community sets high value on women's virginity; these attitudes, in turn, affect access to sexual and reproductive health and rights [37]. Indeed, the availability and accessibility of condoms in the residences and workplaces of migrants should be enhanced, along with on-site free distribution. Further study may be needed to identify the culturally appropriate channel for condom distribution with easy access among those who fill taboo to buy condom. Also, increasing the promotion of condoms for family planning, not only for disease prevention, could hopefully reduce the negative perception of condoms within the Myanmar migrant community.

### Risky sexual behaviors

In the study, 85.3% of the respondents engaged in protective sexual behaviors. The study's findings may differ to a certain point, depending on living conditions, the jobs employed by Myanmar migrant workers, and the gender of the respondents in this study. As explained in the interview that Myanmar migrants in Samut Sakhon still maintain their tradition of the way they live in Myanmar here in Thailand. Being in a group of migrants from the same country in one area or staying in a group with their relatives are the type of living respondents in our study area, Samut Sakhon Province. This situation is also explained in the Report for PHAMIT Project [38], that overcrowded living condition is common in Mahachai, Samut Sakhon Province. Most two-person rooms can accommodate ten or more individuals. Families typically share a room with at least one other family; unmarried or single individuals of the same sex stay together, sometimes dividing the room between day and night shifts. Therefore, staying in a condition where everyone knows each other will give them pressure to behave properly.

As sex is a taboo topic to discuss in Myanmar culture, especially among women, and it may lead them to an uncomfortable situation on answering the questionnaire. Moreover, a study among Myanmar migrant workers in Ranong [39] found that respondents who worked in factory labor had safer sex behaviors compared to other job types. In this study, more than half of the respondents (62.8%) were female, and 75.5% of respondents worked as factory workers, likely influencing the result. In addition, as explained in the method part, we did our survey along with regular blood screening from Samut Sakhon Hospital. Most respondents had been registered by the staff since they regularly visited the hospital. Therefore, we assumed that most respondents were Myanmar migrant workers with legal status or registered. Having a legal status might impact their sexual behavior because they would have health screening, including HIV and syphilis, every time they would like to extend their work permit.

This study also revealed that 90.5% of respondents had one sexual partner, and 2% had sex with a prostitute. Nearly three-quarters, 72.9%, of respondents were married might play a role in this result. The in-depth interview respondents reported that they valued monogamy and they maintained this value even after migrating to Thailand. A study from Hounnaklang et al. (2021) found that most male and female Myanmar workers in Surat Thani, Thailand stated that they did not engage in sexually risky conduct since they were required to live with their spouses most of the time, with no night outs or switching partners. They felt that spending money on parties or prostitutes was wasteful and inappropriate. Also, they believed that condom use was repugnant and that condoms were filthy, hence labeling condom users as promiscuous and polygamous, which is contrary to their culture and tradition [31]. Clearly, this kind of attitude among Myanmar migrant workers can be attributed to their deeply ingrained traditions and inherited societal beliefs. However, a report by Lyttleton (2014) about sexual behaviors among Burmese migrant in Mae Sot showed the contradicted result as they changed their partner easily referring as "Mae Sot love lasts ten minutes" [40]. We speculated that the disparity between a study in Mae Sot and our study may be due to geo-economic activities as Mae Sot is a major economic hub with high trade activities with Myanmar and high population mobility, while most migrant workers in Samut Sakhon migrated with purpose to stay long-term with stable employment; therefore, they brought a whole family, and they could maintain family cohesion and normative expectation toward monogamy. Nonetheless, the further study is needed to validate this speculation. In this study, having two or more sexual partners and having sex with a prostitute were significantly associated with unprotected sexual behavior, which contrasted with the findings from a study among adolescents young adults and older adults in Chiang Mai in which people with multiple partners were more likely to report regular condoms use [41]. Previous studies also revealed that a longer duration of stay in Thailand was statistically associated with having multiple sex partners, visiting sex workers [23], and the practice of unsafe sex with sex workers [39]. However, in this study, 45.9% of the respondents have lived in Thailand for 1–5 years or even more than five years (43.3%), the length of stay among Myanmar migrant in Thailand did not show significant association with unprotective sexual behavior.

We found 28% of the proportion of migrants still have no insurance. A study by Decharatanachart (2021) in Samut Sakhon also showed alarming high of migrant workers’ dependence (98.2%) had no health insurance with the main reason of not buying health insurance was insufficient household income [42] Health authorities should emphasize health education and services to maintain and improve safe sex behavior in this area.

Lastly, 87.8% of the respondent had never used a condom in this study was consistent with previous studies among Myanmar migrant workers conducted elsewhere in Thailand [43], such as in Bangkok [41] and Chiang Mai [44]. This shows a lack of condom use by migrants is a long-established and pervasive tendency that needs to be addressed by health authorities regarding all the factors mentioned above.

### Limitations

Some limitations to this study exist. First, we conducted the study among a community of migrants in only one province; thus, our results cannot be generalized to all Myanmar migrant communities across Thailand. Second, our purposive sampling could have affected the representativeness of the qualitative study sample. For instance, only one man participated in an in-depth interview, this study was likely to represent the women’ perspectives which could provide some insights into the reasons underpinning the STIs/HIV preventive practices. We recommend further study to focus more on male participants.

## Conclusion

We aimed with this study to explore the factors influencing HIV and syphilis preventive behaviors among Myanmar migrants in Samut Sakhon, Thailand. Based on multivariate regression analysis, respondents' income expenses-balance, number of sexual partners, and ever having sex with a prostitute were the most significant quantitative contributors, and in the qualitative analysis, knowledge, understanding, beliefs, culture, community, the environment, and condom perceptions also related to the low utilization of condoms in a Myanmar immigrant community in Samut Sakhon Province, Thailand.

Health education, including knowledge of STIs and condom use, is needed to improve the understanding of the diseases and the importance of safe sex. In addition, health education for family planning should be offered simultaneously to increase the use of condoms as a family planning method. Hopefully, this will lead to the promotion of condoms in terms of family planning, not only for disease prevention, and hopefully could reduce the negative perception of condoms in the Myanmar community. Moreover, the availability and accessibility to condoms in migrants' living places or workplaces should be improved by providing condom-finding machines focused on sites that migrants could access without threat, including on-site free distribution. Organizations working on the health of migrant workers should provide easy accessible information about health insurance benefits to migrant population with a variety of channels to communicate to different migrant groups in Thailand.

## Data Availability

The data supporting this study's findings are available on request from the corresponding author. The data are not publicly available due to privacy or ethical restrictions.
